# The long-term effectiveness and tolerability of PCSK9 inhibitors in a large cardiovascular tertiary centre: A retrospective cohort analysis

**DOI:** 10.21542/gcsp.2026.8

**Published:** 2026-02-28

**Authors:** Mahmud Tawil, Fatima Omar, Solith Senanayake, Michelle Cowdry, Winston Banya, Emma Neves, Charis Browne, Wala Mattar, Richard Grocott-Mason, Mahmoud Barbir

**Affiliations:** 1 Harefield Hospital, Guy’s and St Thomas’ NHS Foundation Trust, London, United Kingdom; 2 Magdi Yacoub Institute, Harefield, Middlesex, United Kingdom; 3 Wexham Park Hospital, Frimley Health NHS foundation Trust, Slough, United Kingdom

## Abstract

**Background:** Proprotein convertase subtilisin/kexin type 9 inhibitors (PCSK9i) are effective LDL-cholesterol–lowering agents used in patients with familial hypercholesterolaemia (FH) and/or very high cardiovascular risk on maximal lipid-lowering therapy. Real-world long-term outcome data from routine clinical practice is of great value but to date there has been scarce data published from within the United Kingdom.

**Methods:** We performed a retrospective analysis of 364 patients treated with PCSK9 inhibitors in a single tertiary centre lipid clinic. Demographic, clinical, lipid and outcome data were extracted from a prospectively maintained database. Baseline was defined as the value immediately prior to PCSK9i initiation; “current” values represent the most recent measurement on therapy. Descriptive statistics are reported; no formal hypothesis testing was performed.

**Results:** Mean age at initiation was 62.2 ± 14.8 years; 57% were male. FH was common: 43.6% had primary heterozygous FH and 3.2% had primary homozygous FH. A further 39.7% had non-FH high or very-high cardiovascular risk despite maximal lipid-lowering therapy. Hypertension was present in ∼38%, diabetes in ∼18%, and ∼22% were current or ex-smokers. At least 42% of patients had documented cardiovascular disease events. Baseline mean LDL-cholesterol was 5.61 ± 1.81 mmol/L (*n* ≈ 360). On treatment, mean LDL-cholesterol fell to 2.37 ± 1.55 mmol/L, corresponding to a mean absolute reduction of 3.26 mmol/L and a mean percentage reduction of 58.7 ± 20.5% (*n* = 352 with paired values). Total cholesterol decreased from 7.86 to 4.56 mmol/L on average. Median follow-up on PCSK9i was approximately 5 years (mean 5.0 ± 1.9 years). At latest follow-up, 304/364 patients (83.5%) remained on PCSK9i. Documented side-effects potentially attributable to PCSK9i occurred in 57 patients (15.7%), most commonly non-specific musculoskeletal symptoms or back pain; discontinuation for adverse events appeared infrequently in the dataset. Major adverse cardiovascular events (MACE) or cardiovascular procedures after PCSK9i initiation were not reliably recorded in in the dataset and therefore the only conclusions that can be made are with regards to lipid lowering effectiveness and tolerability.

**Conclusions:** In this real-world tertiary lipid clinic cohort, PCSK9 inhibitors produced large, durable reductions in LDL-cholesterol of around 60% over a median of ∼5 years, with high treatment persistence and a minimal rate of largely non-serious reported side-effects. The observational design, incomplete event coding and lack of a control group limit inference regarding cardiovascular outcomes, but the lipid-lowering effectiveness is consistent with clinical trial data.

## Introduction

Coronary risk prevention has been at the forefront of research targets for several decades and reduction of low-density lipoprotein cholesterol (LDL-C)^1^ has been effectively achieved with oral medications such as HMG-CoA reductase inhibitors (Statins) and cholesterol absorption inhibitors (Ezetimibe)^2^. Proprotein convertase subtilisin/kexin type 9 inhibitors (PCSK9i) have been utilised as an alternative or adjunctive therapeutic tool in the management of cardiovascular diseases, especially in patients with familial hypercholesterolemia, patient’s intolerant to standard oral therapy, patients unable to achieve (LDL-C) targets on oral therapy alone and those considered at high risk of cardiovascular events^3^.

**Figure 1.**
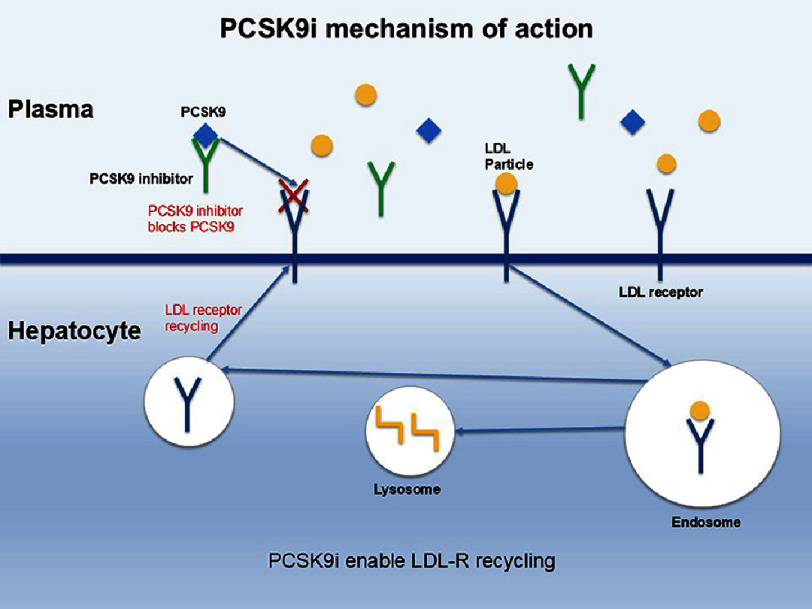
Mechanism of action of PCSK9 inhibitors.

In landmark randomized controlled trials such as FOURIER^4^ and ODYSSEY^5^, PCSK9i administration demonstrated substantial reductions in LDL-C levels (Figure 1). These trials consistently reported significant reductions in LDL-C as well as major adverse cardiovascular events (MACE), including myocardial infarction, stroke, and cardiovascular mortality, compared to standard therapy alone.

Several real-world clinical observational studies have been published from other countries^6^ which support the beneficial effects of PCSK9 inhibition on cardiovascular outcomes. However, only a small amount of data has been published from centres in the United Kingdom^7^.

**Figure 2.**
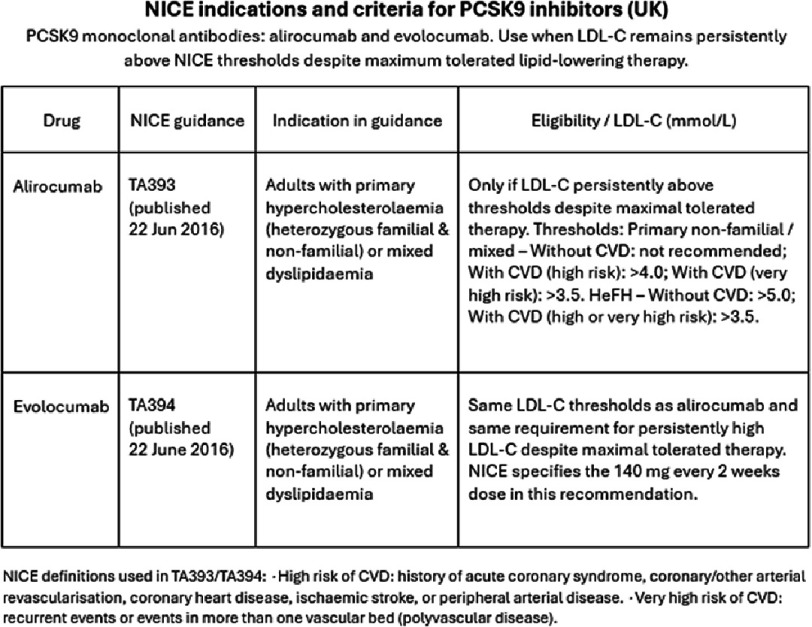
NICE indicators and criteria for PCSK9 inhibitors (UK).

NICE guidance in the United Kingdom (Figure 2) recommends the use of PCSK9 inhibitors for the following indications^8^

This differs from the latest ESC guidelines which were published in 2019 and updated in 2025 which declare that PCSK9i combination use is a Class 1 A recommendation as secondary prevention in patients at very high risk on maximum tolerated dose of Statin and Ezetimibe and not achieving LDL targets, without curtailing their use with a minimal LDL-C level, as noted in the NICE guidance. Those deemed very high risk include patients with documented atherosclerotic cardiovascular disease, diabetes mellitus with end organ damage, type 1 diabetes mellitus with long duration >20 years and severe CKD with an estimated glomerular filtration rate <30ml/min/1.73m^2^. ^9^

However, as with all medications there are various concerns including long-term safety profiles, side effects, cost, and the emergence of other lipid lowering treatment.

The aim of this study is to demonstrate and reflect the experience of a large and current cohort of PCSK9i at a single high volume tertiary cardiovascular centre and compare efficacy and tolerability with the original studies as well as other centres.

## Methods

### Study design and setting

This was a retrospective observational cohort study of all patients recorded at Harefield Hospital which runs a large preventative cardiology service within the Guys and St Thomas’s NHS trust in the United Kingdom. We started an injectable lipid lowering service in 2016 within the Department, mainly run by Clinical Nurse Specialists with support from specialist pharmacists and supervised by NHS Consultant Cardiologist. All patients included received education on the treatment and received at least one PCSK9 inhibitor prescription at the onset. All information was relayed to primary care physicians as per NICE guidance. Data was extracted from the anonymised database, which aggregates patients treated with Evolocumab and Alirocumab.

### Data extraction and cleaning

The following variables were used:

• **Demographics:** Date of birth, age, sex.
• **Clinical indication for PCSK9i:** As recorded in the “Indication” field, e.g.,
  ∘ “1. Homozygous FH on max tolerated”
  ∘ “2. Heterozygous FH on max tolerated”
  ∘ “3. FH Heterozygous with CVD on max tolerated”
  ∘ “5. Non FH high risk on max tolerated”
  ∘ “6. Non FH Very high risk on max tolerated”
• **Therapy details:** Date of initiation of first PCSK9i, length of treatment (years), dose field (e.g., 140 mg, 75 mg).
• **Concomitant therapy:** Other lipid medications (statin, ezetimibe etc.).
• **Lipid parameters:**
  ∘ Baseline (“pre”): LDL-cholesterol, total cholesterol, HDL-cholesterol, t.
  ∘ Latest “current” values on PCSK9i.
• **Comorbidities and risk factors:** Hypertension (HTN), diabetes mellitus (DM), smoking status.
• **Outcomes:**
  ∘ “Still on treatment” (treatment persistence).
  ∘ “Reason for stopping” (if applicable).
  ∘ “Side effects reported (Any PCSK9).”

Lipid parameters were stored as text and converted to numeric values (mmol/L). Descriptive statistics are dominated by the remainder of the dataset.

### Statistical analysis

This is purely descriptive. Continuous variables are summarised using mean ± standard deviation (SD) and, where helpful, median and interquartile range (IQR). Categorical variables are summarised as counts and percentages.

LDL-cholesterol response was assessed in the subset of patients with both a documented baseline LDL and a “current” LDL on treatment (*n* = 352). For each such patient we calculated:

• Absolute change in LDL (mmol/L).
• Percentage change in LDL relative to baseline.

No imputation was performed for missing values, and no formal between-group comparisons or survival analyses were undertaken.

## Results

### Baseline characteristics

A total of 364 patients received PCSK9 inhibitor therapy and were included in the analysis.

• Age: Mean age at initiation was 62.2 ± 14.8 years (range 16–86 years).
• Sex: Male: 219 (58.7%), Female: 154 (41.3%)

### Type of PCSK9i

• Evolocumab: 231 patients
• Alirocumab: 133 patients

**Figure 3.**
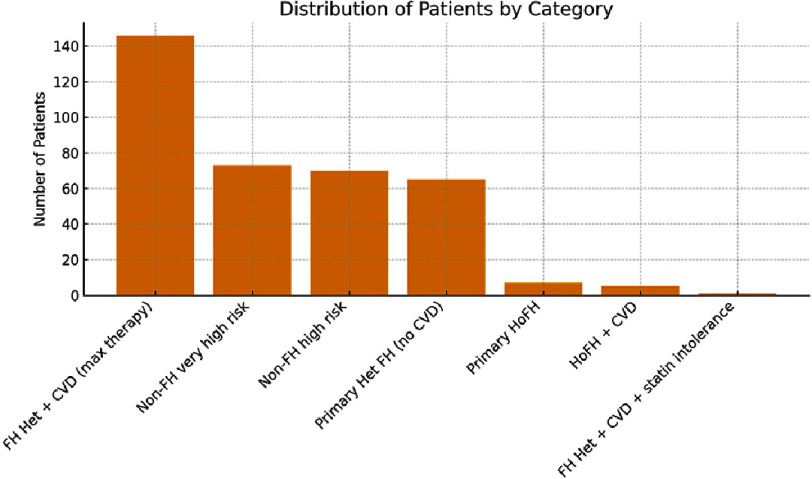
Approximately 61.5% of the cohort had a diagnosis consistent with familial hypercholesterolaemia (heterozygous or homozygous), and a large proportion had established cardiovascular disease and/or very high overall risk. .

**Indication for PCSK9 inhibitor (Figure 3)**:

• FH Het with CVD on maximal therapy: 146 patients (40.1%)
• Non-FH very high risk on maximal therapy: 73 (20.0%)
• Non-FH high risk on maximal therapy: 70 (19.2%)
• Heterozygous FH on maximal therapy (no CVD coded): 65 (17.9%)
• Homozygous FH on maximal therapy: 7 (1.9%)
• FH homozygous with CVD: 5 (1.4%)
• FH Het with CVD and statin intolerance: 1 (0.3%)

### Comorbidities and risk factors

Using the normalised categories:

**Figure 4.**
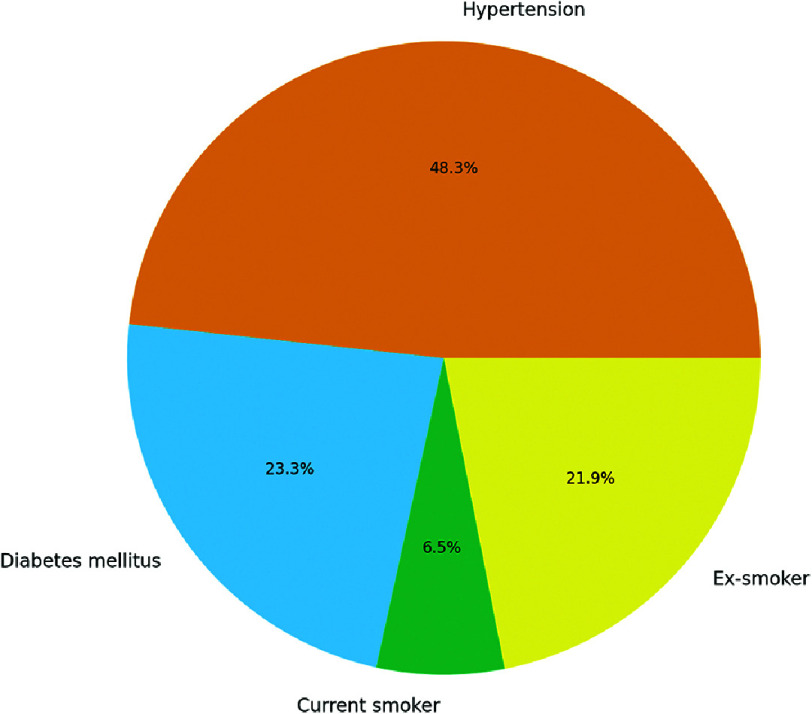
Comorbidities and risk factors.

• Hypertension: 141/364 (38.7%) had documented HTN.
• Diabetes mellitus: 68/364 (18.7%) had diabetes.
• Smoking status: Current smoker: 19/364 (5.2%), ex-smoker: 64/364 (17.6%)

These figures confirm a population at high or very high cardiovascular risk, consistent with guideline indications for PCSK9i (Figure 4).

### Treatment exposure

The recorded length of treatment (years) was available in 344 patients:

• Mean duration: 5.0 ± 1.9 years
• Median: 5.0 years (IQR ≈ 4–6 years)
• Range: 0–9 years

### Lipid-lowering effectiveness

#### Baseline lipid profile

Among patients with available data:

• LDL-cholesterol (pre-PCSK9i):
  ∘ *n* = 360
  ∘ Mean: 5.61 ± 1.81 mmol/L
• Total cholesterol (pre):
  ∘ *n* = 363
  ∘ Mean: 7.86 ± 2.11 mmol/L
• HDL-cholesterol (pre):
  ∘ *n* = 359
  ∘ Mean: 1.96 mmol/L (SD heavily influenced by a small number of outliers).

These values reflect a population with markedly elevated LDL-cholesterol despite “maximal tolerated” background lipid-lowering therapy (statins and often ezetimibe), consistent with severe FH and/or very high-risk primary/secondary prevention.

#### On-treatment (current) lipid profile

Latest recorded values while on PCSK9i:

• LDL-cholesterol (current):
  ∘ *n* = 355
  ∘ Mean: 2.37 ± 1.55 mmol/L
• Total cholesterol (current):
  ∘ *n* = 358
  ∘ Mean: 4.56 ± 1.71 mmol/L
• HDL-cholesterol (current):
  ∘ *n* = 354
  ∘ Mean: 1.44 ± 0.64 mmol/L

#### Paired LDL-cholesterol response

In the subset of 352 patients with both baseline and current LDL-cholesterol values:

• Absolute change in LDL-cholesterol:
  ∘ Mean change: −3.26 ± 1.60 mmol/L
  ∘ Median change: approximately −3.05 mmol/L
  ∘ IQR: about −3.89 to −2.28 mmol/L
• Percentage change in LDL-cholesterol:
  ∘ Mean: –58.7 ±  20.5%
  ∘ Median: approximately –60.5%
  ∘ 25th–75th percentile: roughly –73% to –47%
  ∘ Minimum observed: around –96%
  ∘ Maximum observed: one positive outlier (≈ +71%), likely representing either non-adherence, change of background therapy, or data inconsistency.

Taken together, this suggests that in real-world practice PCSK9 inhibitors achieved approximately 60% mean reductions in LDL-cholesterol, with most patients experiencing a 50–70% reduction, consistent with trial data.

We did not formally stratify LDL response by indication (FH vs non-FH) or by molecule/dose in this summary, but such subgroup analyses could be performed from the same dataset if needed.

### Treatment persistence

• On treatment 304/364 (83.5%)
• Off treatment 49/364 (13.5%)
• Remaining entries indicate that they transferred to a different trust or abroad and were not forcibly recoded but are a small minority.

Thus, most patients remained on PCSK9i at their latest recorded visit, despite mean exposure of ∼5 years. This suggests good long-term persistence in this highly selected tertiary-care population. Reasons for stopping (where recorded) included relocation, loss to follow-up, occasional clinical decisions such as goal achievement or adverse effects and a small number failed to achieve the 30% reduction in LDL-cholesterol from baseline, required for ongoing funding.

### Safety and tolerability

#### Reported side-effects

• Any side-effect recorded: 57/364 (15.7%)
• No side-effects recorded: about 84%

Commonly described symptoms (often in very small numbers each) included:

• Muscle aches
• Back pain
• Flu-like symptoms
• Vivid dreams
• Fatigue, “tiredness”
• Occasional more serious entries such as “Pancreatitis, high CK and ALT”, although it is not possible from this dataset alone to confirm causality or temporal relationship.

Not all recorded symptoms necessarily led to discontinuation, and the structure of the dataset does not allow us to link each adverse effect entry to “Reason for stopping” with certainty. Overall, however, the rate of recorded adverse effects appears modest, and most patients did not have any side-effects documented.

#### Major adverse cardiovascular events (MACE) and procedures

The “MACE since treatment” field is free-text and includes entries such as:

• “MI with PCI to LAD”
• “Stroke 2018”, “TIA”
• “CABG 2016/2020/2021”
• “TAVI 2021”
• “Intervention for PVD 2022”
• Several entries of “RIP” (deaths) with brief descriptions

#### MACE or cardiovascular procedure

• Timing of events relative to start of PCSK9i is not systematically coded (some events appear to pre-date PCSK9i and may have been copied into this field).
• The dataset does not clearly distinguish between outcomes and history in every case.
• There is no denominator in person-years, and no comparator group, so we cannot derive event rates or relative risk reductions.

Therefore, MACE data was not included and cardiovascular events and interventions continued to occur in a high-risk cohort over several years, as expected, and therefore not as a measure of PCSK9i efficacy for event prevention.

## Discussion

In this retrospective, real-world cohort from a tertiary lipid clinic, PCSK9 inhibitor therapy was associated with:

1. **Large and sustained LDL-cholesterol reductions** of approximately 59% on average, with median reductions around 60%, from very high baseline LDL levels typical of severe FH and high-risk non-FH patients.
2. **High treatment persistence**, with over 80% of patients still on PCSK9i after a mean of ∼5 years.
3. **A mild rate of reported side-effects** (∼15%), mostly non-serious symptoms such as musculoskeletal complaints or back pain, with relatively few clearly severe adverse events recorded as potentially drug related.
4. **Ongoing cardiovascular events including cardiac procedures** in about 15% of the cohort, reflecting the extremely high baseline risk, which advances the discussion on the current limitations set in the NICE guidelines on PCSK9i use in very high-risk groups as opposed to the ESC guidelines. However we accept that we are without sufficient detail to infer any effect of PCSK9i on event rates from this data set.

### Comparison with trial and registry data

While this analysis is not linked to external literature in a formal way, the observed ∼60% reduction in LDL-cholesterol is broadly consistent with the magnitude of LDL-lowering reported in major PCSK9i trials^10,11^. The high prevalence of FH and established ASCVD, as well as the presence of hypertension, diabetes and smoking, mirrors typical tertiary lipid clinic populations.

The relatively high long-term treatment persistence may reflect:

• Patient selection (referral to specialist clinic).
• Regular follow-up and support.
• Education and care provided by Specialist nursing team
• The subcutaneous dosing schedule, which, while more invasive than oral therapy, is infrequent (every 2–4 weeks) and may facilitate adherence.

### Clinical implications

From a practical standpoint, these data support the following messages for clinicians managing similar patients:

• **PCSK9 inhibitors deliver robust LDL-cholesterol lowering in ‘real-world’ practice**, comparable to that seen in clinical trials, in both FH and high-risk non-FH patients.
• **Most patients tolerate PCSK9i well over several years**, with only a minority reporting side-effects, and relatively few needing to discontinue therapy solely for tolerability reasons (inferred from the combination of high persistence and low side-effect documentation).
• Given that many patients in this cohort likely had baseline LDL-cholesterol far above guideline targets, the achieved on-treatment mean LDL around 2.4 mmol/L represents a clinically meaningful improvement, though not necessarily target attainment for all, especially in secondary prevention where targets may be ≤1.4 mmol/L depending on the guideline.

## Limitations

This analysis has several important limitations inherent to the dataset:

1. Retrospective, observational design with no control group, preventing causal inference about cardiovascular outcomes.
2. Data quality and completeness:
  ∘ Not all patients have complete lipid panels at both baseline and follow-up.
  ∘ Smoking status, HTN and DM coding is not fully standardised.
3. Outcome coding:
  ∘ Timing relative to PCSK9i initiation is often unclear.
  ∘ No adjudication of event type or cause of death is available.
4. Confounding factors:
  ∘ Background lipid-lowering therapy (statin/ezetimibe changes) and other risk-modifying interventions over time are not systematically captured.
  ∘ Adherence to PCSK9i is not directly measured (only inferred from continued prescribing).
5. Generalisability:
  ∘ Single-centre, tertiary-care cohort, likely enriched for complex FH and very high-risk patients. Results may not generalise to lower-risk or primary-care populations.

## Conclusions

In a real-world tertiary lipid clinic cohort of 364 patients, predominantly with familial hypercholesterolaemia and/or very high cardiovascular risk already on maximal lipid-lowering therapy, PCSK9 inhibitors produced large, durable reductions in LDL-cholesterol (around 60% on average) over a mean follow-up of ∼5 years.

Treatment persistence was high (>80% remained on therapy at last follow-up), and recorded adverse effects were relatively infrequent and generally non-serious, suggesting that PCSK9 inhibitors are both effective and well-tolerated in this setting.

Although cardiovascular events and procedures continued to occur in this very high-risk cohort, the observational design and limitations of outcome coding prevent any firm conclusions about event reduction. Nevertheless, the lipid-lowering effectiveness demonstrated here is consistent with the expected biological impact of PCSK9 inhibition and supports their ongoing use in appropriately selected patients. The magnitude of reduction in LDL-cholesterol and excellent tolerability one would expect a significant reduction in cardiac events as published in other studies. We believe there are limitations placed by current UK guidelines on which patients can benefit from these novel medications, particularly those in the secondary prevention group with high-risk features and unable to achieve LDL-C targets on maximum oral therapy. LDL-cholesterol is high at 3.5 mmol/L and inconsistent with new therapies at a lower level of 2.6 mmol/L. Although our data and experience can only provide evidence of excellent and sustained lipid lowering ability of the medication as well as the general tolerability with small reporting of side effects. We would propose that in combination with other data sets around the world as well as the original trial data which additionally shows meaningful reduction in major adverse cardiovascular events^12^, that this would support the foundation for an update to the UK NICE guidance to favourably adopt the ESC guidelines.
